# High infant mortality, eschar absence, and universal CNS involvement in scrub typhus-associated HLH: a case series and narrative synthesis of 91 pediatric cases

**DOI:** 10.1186/s41182-026-00988-6

**Published:** 2026-05-30

**Authors:** Shuanglinzi Deng, Chun Yang, Shuangyu Yang, Tianyu Yang, Yuanyuan Li, Xia Wang, Jing Peng, Xiaolu Deng

**Affiliations:** 1 Department of Pediatrics, Xiangya Hospital of Central South University, Chang Sha, 410008 Hunan Province China; 2 Department of Pediatrics, Pu‘er People’s Hospital, Pu’er, 665099 Yunnan Province China; 3 Department of Respiratory Medicine, National Key Clinical Specialty, Branch of National Clinical Research Center for Respiratory Disease, Xiangya Hospital, Central South University, Changsha, 410008 Hunan China; 4 National Clinical Research Center for Geriatric Diseases, Xiangya Hospital, Changsha, 410008 Hunan China

**Keywords:** Scrub typhus, Hemophagocytic lymphohistiocytosis, Infant, Molecular diagnostics, Mortality

## Abstract

**Background:**

Scrub typhus-associated hemophagocytic lymphohistiocytosis (HLH) is a life-threatening complication of Orientia tsutsugamushi infection. Its clinical features in young infants remain poorly characterized, despite evidence that immune immaturity and atypical presentations may confer particular vulnerability in this age group.

**Methods:**

We present a case series of three infants with scrub typhus-associated HLH from Yunnan Province, southwestern China, confirmed by molecular diagnostics. Combined with a pooled analysis of published pediatric cases identified through a structured search of 5 databases from inception to December 2025, we analyzed a total of 91 cases.

**Results:**

Infant mortality was markedly higher than that in older children (44.4% vs. 15.9%). Eschar detection was substantially lower in infants than non-infants (22.2% vs. 66.7%), and central nervous system (CNS) involvement was universal among infants (100% vs. 42.9%). In eschar-negative cases, molecular diagnostics were essential for etiological confirmation. All comparisons are exploratory and hypothesis-generating.

**Conclusions:**

Scrub typhus-associated HLH in young infants is associated with high mortality, frequent eschar absence, and universal CNS involvement, representing a clinical profile distinct from older children. In endemic areas, early molecular testing should be prioritized in eschar-negative presentations, and empirical doxycycline initiated promptly, with rifampin considered when CNS involvement is confirmed. Prospective multicenter studies are needed to validate these findings.

**Supplementary Information:**

The online version contains supplementary material available at 10.1186/s41182-026-00988-6.

## Background

Scrub typhus, caused by Orientia tsutsugamushi, is a significant public health threat in the Asia–Pacific region, with an estimated one million cases occurring annually within the endemic “tsutsugamushi triangle” [1]. Transmitted through the bite of infected Leptotrombidium mites, the disease typically presents with fever, rash, and—most pathognomonically—an eschar at the bite site. When identified early, standard antibiotic therapy reliably achieves an excellent prognosis. However, delayed diagnosis, often attributable to limited healthcare access in rural endemic settings, can precipitate devastating multi-organ complications.

Among these, hemophagocytic lymphohistiocytosis (HLH) is the most lethal. HLH is a life-threatening hyperinflammatory syndrome driven by uncontrolled activation of macrophages and cytotoxic lymphocytes, clinically manifesting as persistent fever, hepatosplenomegaly, cytopenias, hyperferritinemia, and hemophagocytosis [2, 3]. Diagnosis is standardized according to the HLH-2004 criteria, which require fulfillment of at least five of eight defined clinical and laboratory features. Secondary HLH can be triggered by infections, malignancies, or autoimmune conditions, and scrub typhus has emerged as an increasingly recognized infection trigger in endemic regions—though the true incidence remains uncertain due to underdiagnosis of both conditions [4, 5].

The diagnostic intersection of scrub typhus and HLH poses challenges. The eschar—the sign that most reliably raises clinical suspicion—is detected in approximately 50–80% of pediatric scrub typhus cases across most series, and may be identified in fewer than 20% of those with concurrent CNS involvement [6–9]. In its absence, serological testing typically requires 7–10 days for antibody development, while molecular diagnostics—though highly informative—remain unavailable in many resource-limited endemic settings [10]. This diagnostic gap is especially consequential in HLH, where a hyperinflammatory state demands urgent intervention and diagnostic delay can prove fatal.

Infants represent a uniquely vulnerable group within this landscape. Although limited outdoor mobility might theoretically reduce mite exposure, peridomestic and even vertical transmission has been documented [11, 12]. The immature infant immune system—characterized by limited natural killer cell function and reduced cytotoxic T-cell activity—may predispose to dysregulated immune activation [13, 14]. Clinically, infant eschars tend to be small and concealed within skin folds, the scalp, or the diaper area, escaping detection even on careful examination. Compounding this, the nonspecific presenting features of severe infection in infancy—irritability, poor feeding, and fever—overlap broadly with common pediatric conditions, frequently delaying consideration of rickettsial disease until critical deterioration has occurred.

Despite these biological and clinical vulnerabilities, the characterization of scrub typhus-associated HLH specifically in young infants remains fragmentary. Over the past decade, case reports from India [15, 16], China [17, 18, 22], South Korea [19, 20], and Thailand [21] have collectively outlined the pediatric clinical spectrum, suggesting favorable outcomes with prompt anti-rickettsial therapy and immunomodulation. However, these studies predominantly involve school-aged children with identifiable eschars. Fewer than 10 infant cases have been documented in the literature to date.

We present three infant cases of scrub typhus-associated HLH from Pu’er City, Yunnan Province—a region in southwestern China bordering Myanmar that represents a previously unreported endemic focus. All three infants were under seven months of age, lacked identifiable eschars, and required molecular diagnostics for etiological confirmation. We complement this case series with a comprehensive pooled analysis of all published pediatric cases, yielding a combined cohort of 91 patients—the largest analysis to date. Our objectives were to: (1) characterize the clinical features, diagnostic approaches, and outcomes of scrub typhus-associated HLH in young infants; (2) describe the pooled clinical profile of pediatric cases; and (3) identify factors associated with mortality in this vulnerable population.

## Methods

### Study design

This study combines a retrospective case series with a narrative synthesis of individual patient data from a systematic search, yielding a combined cohort of 91 pediatric cases. Case presentations adhered to CARE guidelines, and the overall study is reported in accordance with the STROBE checklist for observational studies (Supplementary Table S1). A formal systematic review with meta-analysis was not feasible due to substantial heterogeneity in diagnostic criteria, outcome definitions, and data completeness across included sources, as well as the predominance of case reports and small case series precluding pooled quantitative synthesis. All comparative findings should be considered exploratory and hypothesis-generating pending prospective validation.

### Case definitions for scrub typhus

Patients were classified into two diagnostic categories. Confirmed case: clinical features compatible with scrub typhus plus at least one of: (1) detection of Orientia tsutsugamushi DNA by polymerase chain reaction (PCR) or next-generation sequencing (NGS) from blood, cerebrospinal fluid, or tissue; or (2) seroconversion with a ≥ 4-fold rise in IgG titers between paired acute and convalescent sera. Probable case: compatible clinical manifestations with a clear epidemiological link, supported by a single positive serological result (positive IgM or high IgG titer via ELISA, IFA, or Weil–Felix test) without confirmatory molecular or paired-sera testing.

### Novel case series

Three cases were identified from the Pediatric Intensive Care Unit (PICU) of Pu’er People’s Hospital, Yunnan Province, China, between May and October 2025. Inclusion criteria required: (1) age < 18 years; (2) Orientia tsutsugamushi infection confirmed by molecular methods (PCR or NGS); and (3) fulfillment of HLH-2004 diagnostic criteria (≥ 5 of 8 features: fever ≥ 38.5 °C, splenomegaly, cytopenias affecting ≥ 2 lineages, hypertriglyceridemia > 3.0 mmol/L and/or hypofibrinogenemia < 1.5 g/L, hemophagocytosis in bone marrow or other tissues, low or absent NK cell activity, ferritin ≥ 500 ng/mL, and elevated soluble CD25). Clinical data were extracted from electronic medical records, including demographic characteristics, epidemiological exposure, clinical manifestations, laboratory findings, microbiological results, treatment regimens, and outcomes. This study was approved by the Ethics Committee of Pu’er People’s Hospital, and written informed consent was obtained from all patients' parents.

### Literature search and case identification

A structured literature search was conducted across PubMed, Embase, Web of Science, China National Knowledge Infrastructure (CNKI), and Wanfang Data from inception to December 15, 2025, without language restrictions to identify published pediatric cases with individual patient data available for narrative analysis. Search terms combined concepts for scrub typhus (“scrub typhus”, “Orientia tsutsugamushi”, “tsutsugamushi”), hemophagocytic lymphohistiocytosis (“hemophagocytic lymphohistiocytosis”, “hemophagocytic syndrome”, “HLH”, “macrophage activation syndrome”), and pediatric population (“child”, “infant”, “adolescent”, “pediatric”). Chinese equivalents were used for CNKI and Wanfang. Reference lists of included articles were manually searched for additional studies. The full search strategies are detailed in Supplementary Table S2.

Studies were included if they reported pediatric patients (< 18 years) with scrub typhus-associated HLH, with sufficient data to extract age, sex, treatment, and outcome. Studies were excluded if they reported exclusively adult patients, HLH due to other etiologies, or lacked individual patient data; review articles, editorials, and conference abstracts without original data were also excluded.

Two investigators independently reviewed identified records and extracted data using a standardized form; disagreements were resolved by consensus or referral to a third investigator. Duplicate cases were identified by matching patient demographics, admission dates, and clinical features across publications. For multi-patient series where complete individual-level data were not fully recoverable, available data were extracted to the extent possible, with missing variables recorded as “not reported”.

### Definitions

Infants were defined as children aged < 12 months at admission, per WHO age classifications. Eschar status was classified as present, absent (explicitly documented as), or not reported. CNS involvement included seizures, altered consciousness, meningitis, encephalitis, or cerebrospinal fluid abnormalities. Mortality was defined as death during index hospitalization or within 30 days of admission.

### Statistical analysis

Continuous variables were expressed as median (IQR) and categorical variables as frequencies and percentages. Pre-specified subgroup analyses compared infants versus non-infants and survivors versus non-survivors. For key binary outcomes (mortality, eschar detection, CNS involvement), risk differences (RD) with 95% confidence intervals were calculated. Categorical variables were compared using Chi-square test or Fisher’s exact test; continuous variables using Mann–Whitney *U* test. Given the retrospective, heterogeneous, multi-country nature of the pooled data, all P values are exploratory and should not be interpreted as confirmatory; no adjustments for multiple comparisons were applied. A sensitivity analysis restricted the infant versus non-infant mortality comparison to studies contributing complete individual patient data. All analyses were performed using SPSS version 26.0 (IBM Corp., Armonk, NY, USA).

## Results

### Novel case series: three infant cases of scrub typhus-associated HLH from southwestern China

Between May and October 2025, three infants with scrub typhus-associated HLH were admitted to the PICU of Pu’er People’s Hospital. All were of Lahu ethnicity residing in remote mountainous areas of southwestern China. Clinical characteristics are summarized in Table 1, with detailed laboratory findings in Supplementary Table S3.

**Table 1:** Clinical characteristics of three infant cases of scrub typhus-associated HLH from southwestern China

Characteristic	Case 1	Case 2	Case 3
Demographics			
Age	2 months 7 days	5 months 15 days	6 months 1 day
Sex	Female	Female	Male
Ethnicity	Lahu	Lahu	Lahu
Location	Menglian County	Lancang County	Lancang County
Epidemiology			
Exposure history	Reservoir visit 6 days before onset	Frequent visits to mountainous cornfields	Not documented (rural endemic area)
Clinical presentation			
Fever duration before admission	6 days	11 days	4 days
Eschar	Absent	Absent	Absent
Hepatomegaly	Yes (7 cm below RCM)	Yes (5 cm below RCM)	No
Splenomegaly	Yes (2 cm below LCM)	Yes	Yes (3 cm below LCM)
Respiratory distress	Yes	Yes (ARDS)	Yes
CNS involvement	Suspected	Seizures	Meningoencephalitis
Key laboratory findings			
Platelet nadir (× 10^9^/L)	30	31	19
Hemoglobin nadir (g/L)	72	105	92
Ferritin (ng/mL)	ND	ND	6800 (peak); 628.76 (discharge)
Fibrinogen nadir (g/L)	Low (not specified)	2.1	0.22
Triglycerides (mmol/L)	Elevated	4.33	3.33–4.48
Bone marrow hemophagocytosis	ND	ND	Present
sCD25 (U/mL)	ND	ND	25,714
NK cell activity	ND	ND	Decreased (4.18%)
HLH-2004 criteria met	5/8	Probable HLH ^†^	7/8
Etiological diagnosis			
Diagnostic method	qPCR (whole blood)	tNGS (throat swab)	tNGS (CSF, blood) + PCR
Timing of confirmation	Posthumous (Day 3)	Posthumous (Day 2)	Day 4 of hospitalization
Co-infections	None identified	CMV, Rhinovirus B, *S. pneumoniae*, *S. maltophilia*	EBV, Rhinovirus, Candida albicans
Treatment			
Anti-rickettsial agent	Azithromycin (empirical)	None	Doxycycline + rifampin
Corticosteroids	Methylprednisolone	Methylprednisolone	Methylprednisolone → prednisone taper
IVIG	Yes	Yes	Yes
Cyclosporine	No	No	Yes
Etoposide	No	No	No
Mechanical ventilation	Yes	Yes	Yes
CRRT	Yes	Yes	Yes
Outcome			
Result	Died	Died	Survived
Time to outcome	~ 30 h	~ 19 h	35 days (discharge)
Cause of death	Refractory shock with consumptive coagulopathy	Refractory shock with consumptive coagulopathy	NA

Ferritin, sCD25, and bone marrow examination were not performed in Cases 1 and 2 due to rapid clinical deterioration; ^†^Case 2 classified as probable HLH based on clinical judgment (fever, splenomegaly, thrombocytopenia, hypertriglyceridemia, multi-organ dysfunction in endemic context)

### Case 1

A 2-month-7-day-old female presented with 6 days of fever following reservoir exposure. Despite prior broad-spectrum antibiotics and methylprednisolone at a county hospital, she deteriorated progressively. On admission, she was critically ill with hepatosplenomegaly and petechiae but no identifiable eschar; cerebrospinal fluid (CSF) showed pleocytosis (24 × 10^6^/L) with elevated protein, indicating CNS involvement.

Despite aggressive support—mechanical ventilation, CRRT, corticosteroids, and IVIG—she developed refractory shock with consumptive coagulopathy (severe thrombocytopenia, hypofibrinogenemia, petechiae) and died approximately 30 h after admission. Quantitative PCR on whole blood confirmed Orientia tsutsugamushi infection posthumously.

Five of the eight HLH-2004 criteria were met on available data (fever, splenomegaly, bilineage cytopenias, hypertriglyceridemia, hypofibrinogenemia); bone marrow confirmation, ferritin, sCD25, and NK cell activity could not be obtained due to fulminant deterioration.

### Case 2

A 5-month-15-day-old female, with frequent visits to mountainous cornfields prior to illness, presented with 11 days of fever and new-onset generalized tonic–clonic seizures. On admission she was somnolent and jaundiced, with sluggish pupillary reflexes and increased muscle tone; no eschar was identified. Mechanical ventilation and methylprednisolone pulse therapy were initiated immediately but failed to prevent cardiac arrest on hospital day 2, approximately 19 h after admission. Targeted next-generation sequencing (tNGS) of a throat swab posthumously detected O. tsutsugamushi (1253 reads) alongside co-pathogens (Streptococcus pneumoniae, Rhinovirus B, Cytomegalovirus). Because throat swab is not among the standard specimens specified in our confirmed case definition, Case 2 is classified as probable scrub typhus, supported by the high-read tNGS detection, compatible clinical presentation, and clear epidemiological exposure.

HLH was diagnosed on clinical judgment: documented features included fever, splenomegaly, thrombocytopenia, and hypertriglyceridemia in the context of overwhelming multi-organ dysfunction; fibrinogen (2.1 g/L) did not meet the hypofibrinogenemia threshold, and bone marrow examination, ferritin measurement, sCD25, and NK cell activity were unobtainable prior to cardiac arrest.

### Case 3

A 6-month-1-day-old male presented with 4 days of fever, respiratory distress, and petechial rash. No eschar was identified despite thorough examination of the scalp, axillae, and perineum. He fulfilled 7 of 8 HLH-2004 criteria: fever, splenomegaly, bilineage cytopenias, hyperferritinemia (peak 6800 ng/mL), hypofibrinogenemia (nadir 0.22 g/L), hypertriglyceridemia, and bone marrow hemophagocytosis; soluble CD25 was markedly elevated (25,714 U/mL). CSF tNGS detected O. tsutsugamushi (248 reads), and peripheral blood tNGS confirmed persistent infection; co-infections included EBV, Rhinovirus, and Candida albicans.

Treatment comprised doxycycline with rifampin for CNS penetration, methylprednisolone (tapered to oral prednisone), cyclosporine, IVIG. Fever resolved and HLH parameters normalized; the patient was discharged after 35 days with intact neurological function.

### Summary of novel cases

Three observations emerged from this series. First, no infants had an identifiable eschar, making molecular methods (qPCR or tNGS) indispensable for etiological confirmation. Second, both fatal cases died within 19–30 h of admission—before diagnosis was established—underscoring the necessity of early empirical treatment. Third, the sole survivor differed from the fatal cases in shorter pre-admission fever duration (4 vs. 6–11 days), timely molecular diagnosis (day 4 of hospitalization), combination anti-rickettsial therapy with CNS-penetrating agents (doxycycline plus rifampin), and addition of cyclosporine. The shared Lahu ethnicity and remote residence of all three cases likely contributed to delayed presentation in the fatal cases.

### Baseline characteristics of pooled cases

Individual patient data for all 91 cases are presented in Table 2; baseline characteristics are summarized in Table 3. The median age was 60 months (IQR 36.0–84.0); 9 cases (9.9%) were infants under 12 months with the youngest being a 13-day-old neonate with suspected vertical transmission [11]. Cases were predominantly from China (46.2%) and India (48.4%), with the majority originating from southwestern and southern China (Sichuan, Yunnan, Guangdong, Hong Kong), and northern/eastern India. No identifiable exposure history was recorded in 50.5% of cases overall, and in 44.4% of infants, suggesting that peridomestic transmission may be underrecognized in this age group.

**Table 2:** Individual patient characteristics of 91 pediatric scrub typhus-associated HLH cases

Case ID	Source (author, year)	Country	Age-months	Sex	Key symptoms/signs			Etiological confirmation	Outcome
					Fever	Fever-days (prior to admission)	Eschar	Method	
1–18	Parajuli B, 2021 [15]	India	81 (55–127) ^*^	Male: 12; female: 6	Yes	NR	NR	ELISA	Death: 9 (50%); survived 9 (50%)
19	Wu, 2025 [22]	China	106	Male	Yes	4	Yes	mNGS	Survived
20	Han et al., 2012 [19]	South Korea	108	Female	Yes	**7**	Yes	IFA	Survived
21	Kwon et al., 2013 [19, 20]	South Korea	8	Male	Yes	10	Yes	IFA	Survived
22	Pazhaniyandi et al., 2015 [19, 23]	India	2	Male	Yes	5	No	ELISA	Survived
23	Zhou et al., 2016 [17]	China	72	Male	Yes	7	Yes	Weil–Felix test	Survived
24	Zhou et al., 2016 [17]	China	48	Female	Yes	9	Yes	Weil–Felix test	Survived
25	Zhou et al., 2016[17]	China	36	Female	Yes	8	Yes	Weil–Felix test	Survived
26	Jin et al., 2016 [18]	China	8	Male	Yes	9	Yes	Weil–Felix test/or IFA	Death
27	Jin et al., 2016 [18]	China	15	Female	Yes	4	Yes	Weil–Felix test/or IFA	Survived
28	Jin et al., 2016 [18]	China	84	Male	Yes	12	Yes	Weil–Felix test/or IFA	Survived
29	Jin et al., 2016 [18]	China	84	Female	Yes	9	Yes	Weil–Felix test/or IFA	Survived
30	Jin et al., 2016 [18]	China	132	Male	Yes	7	Yes	Weil–Felix test/or IFA	Survived
31	Jin et al., 2016 [18]	China	84	Male	Yes	7	Yes	Weil–Felix test/or IFA	Survived
32	Jayakrishnan et al., 2011 [24]	India	60	Female	Yes	6	Yes	Weil–Felix test/or ELISA	Survived
33–41	Lin et al., 2019 [25]	China	120(11–204)^†^	Male: 5; female: 4	Yes	NR	Yes	Weil–Felix test/or IFA	Survived
42	Gupta et al., 2021 [26]	India	168	Female	Yes	10	NR	ELISA	Survived
43–60	Basu A, 2021 [16]	India	35.3 ± 44.8(1–144)^†^	Male: 11; female: 7	Yes	9.2	6 (33%)	ELISA	Death: 2 (11%); survived 16 (89%)
61	Agrwal S, 2019 [27]	India	8	Male	Yes	10	No	Weil–Felix test, ELISA	Death
62–77	Jin Y-M, 2018 [28]	China	56.3 ± 42.24^†^	Male: 6; female: 10	Yes	13	9.19 ± 3.17	Weil–Felix test	Death: 1 (6.25%); survived 15 (93.75%)
78	Luo Y, 2025 [11]	China	0.43	Female	Yes	3	No	PCR, Weil–Felix test, ELISA	Survived
79	Kunanitthaworn N, 2025 [21]	Thailand	23	Female	Yes	11	No	IFA	Survived
80	Kunanitthaworn N, 2025 [21]	Thailand	14	Female	Yes	21	Yes	IFA	Survived
81	Kunanitthaworn N, 2025 [21]	Thailand	20	Female	Yes	7	No	IFA	Survived
82	Jian H, 2024 [29]	China	72	Female	Yes	7	No	mNGS and qPCR	Survived
83	Jian H, 2024 [29]	China	120	Female	Yes	6	No	mNGS and qPCR	Survived
84	Fung RCM, 2022 [30]	China	84	Male	Yes	7	No	IFA	Survived
85	Sankhyan N, 2014 [31]	India	84	Male	Yes	7	UK	ELISA	Survived
86	Sankhyan N, 2014 [31]	India	108	Female	Yes	10	NR	ELISA	Survived
87	Sankhyan N, 2014 [31]	India	78	Female	Yes	8	NR	ELISA	Death
88	Sahu SK, 2021 [32]	India	3	Female	Yes	10	No	ELISA	Survived
89	Case1	China	2.2	Female	Yes	6	No	qPCR	Death
90	Case2	China	5.5	Female	Yes	11	No	tNGS (throat swab)	Death
91	Case3	China	6	Male	Yes	4	No	tNGS (CSF), Specific nucleic acid test	Survived

^*^Data presented as median (IQR). †Data presented as mean ± SD or mean (range)

**Table 3:** Baseline characteristics of pediatric scrub typhus-associated HLH cases

Characteristic	All cases (*N* = 91)	Infants (< 12 months) (*N* = 9)	Non-infants (≥ 12 months) (*N* = 82)
Age (months)			
Median (IQR)	60.0 (36.0–84.0)	5.5 (2.2–8.0)	72.0 (48.0–96.0)
Range	0.43–168	0.43–8	12–168
Sex, *n* (%)			
Male	46 (50.5%)	5 (55.6%)	41 (50.0%)
Female	45 (49.5%)	4 (44.4%)	41 (50.0%)
Geographic region, *n* (%)			
China	42 (46.2%)	5 (55.6%)	37 (45.1%)
India	44 (48.4%)	3 (33.3%)	41 (50%)
South Korea	2 (2.2%)	1 (11.1%)	1 (1.2%)
Thailand	3 (3.3%)	0 (0%)	3 (3.7%)
Exposure history, *n* (%)			
Documented exposure	34 (37.4%)	4 (44.4%)	30 (36.6%)
No identifiable exposure	46 (50.5%)	4 (44.4%)	42 (51.2%)
Not reported	11 (12.1%)	1 (11.1%)	10 (12.3%)
Data source, *n* (%)			
Literature cases	88 (96.7%)	6 (66.7%)	82 (100%)
Novel cases	3 (3.3%)	3 (33.3%)	0 (0%)

### Clinical manifestations

Clinical features are presented in Table 4. Fever was universal (100%), with a median pre-admission duration of 7.0 days (IQR 6.0–10.0). Eschar was identified in only 60.9% (42/69) of cases with documented examination; detection was substantially lower in infants than non-infants (22.2% vs 66.7%; exploratory *P* = 0.009). Hepatomegaly and splenomegaly were present in 73.8% and 84.6% of cases, respectively. CNS involvement was documented in 48.1% (37/77) overall but occurred was more frequent in infants (100%, 7/7) than in non-infants (42.9%, 30/70; exploratory *P* = 0.012). ARDS (88.5%), DIC (86.8%), and MODS (82.4%) were common complications, with infants showing higher rates of multi-organ involvement.

**Table 4:** Clinical manifestations of pediatric scrub typhus-associated HLH cases

Clinical feature	All cases	Infants (< 12 months)	Non-infants (≥ 12 months)	RD (95% CI)	Exploratory *P* value*
Fever	91/91 (100%)	9/9 (100%)	82/82 (100%)		–
Fever duration, days, median (IQR)	7.0 (6.0–10.0)	9.0 (5.0–10.0)	7.0 (6.0–9.3)		0.876
Eschar present	42/69 (60.9%)	2/9 (22.2%)	40/60 (66.7%)	− 44.5% (− 72.4 to − 16.6%)	**0.009**
Hepatomegaly	45/61 (73.8%)	7/8 (87.5%)	38/53 (71.7%)		0.672
Splenomegaly	66/78 (84.6%)	7/8 (87.5%)	59/70 (84.3%)		1.000
ARDS	46/52 (88.5%)	7/7 (100%)	39/45 (86.7%)		0.580
CNS involvement	37/77 (48.1%)	7/7 (100%)	30/70 (42.9%)	+ 57.1%^†^	**0.012**
Rash	35/60 (58.3%)	–	–		–
DIC	33/38 (86.8%)	7/8 (87.5%)	26/30 (86.7%)		1.000
Shock	28/30 (93.3%)	–	–		–
MODS	28/34 (82.4%)	5/5 (100%)	23/29 (79.3%)		0.567

^*^P values are exploratory and should not be interpreted as confirmatory, given the retrospective, heterogeneous nature of the pooled data derived from multiple countries and study designs with variable diagnostic criteria and reporting completeness

RD risk difference, 95% CI 95% confidence interval

^†^95% CI not calculable as infant proportion = 1.0; RD presented as point estimate only

Bold marks exploratory P values below 0.05

### Laboratory findings and HLH diagnostic criteria

HLH-2004 criterion fulfillment is summarized in Table 5, with detailed laboratory values in Supplementary Table S4. Bilineage cytopenias were present in 97.8% (89/91) of cases; median platelet nadir was 45 × 10^9^/L overall; lower in infants (30 × 10^9^/L). Hyperferritinemia (> 500 ng/mL) was nearly universal (93.3%, range from 587 to > 19,000 ng/mL). Hypofibrinogenemia (< 1.5 g/L) was present in 73.4% (58/79) and hypertriglyceridemia (> 3.0 mmol/L) in 69.7% (62/89) of cases with available data. Bone marrow hemophagocytosis was demonstrated in 92% (81/88) of examined cases; 3 of 9 infants did not undergo bone marrow examination due to rapid clinical deterioration. Among the minority of cases tested, sCD25 was markedly elevated in all (8367–25,714 U/mL) and NK cell percentage consistently decreased (4.18–15.5%).

**Table 5:** Fulfillment of HLH-2004 diagnostic criteria

HLH-2004 criterion	All cases *n*/*N* (%)	Infants (< 12 months) *n*/*N* (%)	Non-infants (≥ 12 months) *n*/*N* (%)
Cytopenias (≥ 2 lineages)	89/91 (97.8%)	9/9 (100%)	80/82 (97.6%)
Hyperferritinemia (> 500 ng/mL)	83/89 (93.3%)	7/7 (100%)	76/82 (92.7%)
Hypofibrinogenemia (< 1.5 g/L)	58/79 (73.4%)	5/6 (83.3%)	53/73 (72.6%)
Hypertriglyceridemia (> 3.0 mmol/L)	62/89 (69.7%)	6/7 (85.7%)	56/82 (68.3%)
Hemophagocytosis in BM	81/88 (92%)	4/6 (66.7%)	77/82 (93.9%)
Elevated sCD25	4/4 (100%)	1/1 (100%)	3/3 (100%)
Decreased NK cell activity	30/30 (100%)	1/1 (100%)	29/29 (100%)

### Diagnostic methods for scrub typhus

Diagnostic methods are summarized in Table 6. Serological testing predominated, used in 93.4% of cases (85/91), including ELISA (49.5%), Weil–Felix test (40.7%), and IFA (23.1%). Molecular methods were used in only 7.7% cases (7/91) overall, but in 44.4% (4/9) of infants versus 3.7% (3/82) of non-infants. Co-infections were identified in 36.2% (17/47) of cases with available data, most commonly (9 cases); both fatal novel cases harbored multiple co-pathogens detected by tNGS.

**Table 6:** Diagnostic methods for scrub typhus in pediatric HLH cases

Diagnostic method	*n*/*N* (%)
Serological methods	**85/91 (93.4%)**
ELISA	45 (49.5%)
Weil–Felix test	37 (40.7%)
IFA	21 (23.1%)
Molecular methods	**7/91 (7.7%)**
NGS	5 (5.5%)
PCR	5 (5.5%)

Categories not mutually exclusive; some cases used multiple methods. Percentages for specific methods based on 91 cases with available data

Bold marks the two category subtotals (Serological methods; Molecular methods)

### Treatment strategies

Treatment strategies are summarized in Table 7. Doxycycline was the most frequently used anti-rickettsial agent (38.5%), followed by azithromycin (20.9%) and chloramphenicol (14.3%). Among the novel infant cases, only Case 3 received targeted anti-rickettsial therapy (doxycycline plus rifampin); Cases 1 and 2 were treated with empirical antibiotics without rickettsial coverage due to delayed diagnosis. Corticosteroids were administered in 80.0% (56/70) and IVIG in 48% (24/50) of cases with available data. Etoposide was used in only 8.3% (4/48) of cases, yet all 4 patients survived. Mechanical ventilation was required in 53.8% of cases overall, with higher utilization in infants (87.5% vs 49.1%).

**Table 7:** Treatment strategies in pediatric scrub typhus-associated HLH

Treatment	All cases *n*/*N* (%)	Infants (< 12 months) *n*/*N* (%)	Non-infants (≥ 12 months) *n*/*N* (%)
Anti-rickettsial antibiotics			
Doxycycline	35/91 (38.5%)	4/9 (44.4%)	31/82 (37.8%)
Chloramphenicol	13/91 (14.3%)	0/9 (0%)	13/82 (15.9%)
Azithromycin	19/91 (20.9%)	2/9 (22.2%)	17/82 (20.7%)
Other macrolides	2/91 (2.2%)	1/9 (11.1%)	1/82 (1.2%)
Rifampin	2/91 (2.2%)	1/9 (11.1%)	1/82 (1.2%)
Immunomodulatory therapy			
Corticosteroids	56/70 (80.0%)	6/9 (66.7%)	50/61 (82.0%)
IVIG	24/50 (48%)	6/7 (85.7%)	18/43 (41.9%)
Etoposide	4/48 (8.3%)	1/5 (20.0%)	3/43 (7.0%)
Organ support			
Mechanical ventilation	35/65 (53.8%)	7/8 (87.5%)	28/57 (49.1%)
Vasoactive agents	15/91 (16.5%)	1/9 (11.1%)	14/82 (17.1%)
CRRT	12/91 (13.2%)	3/9 (33.3%)	9/82 (11.0%)
Plasma exchange	3/91 (3.2%)	1/9 (11.1%)	2/82 (2.4%)

### Outcomes

Overall mortality was 18.7% (17/91), with 74 patients (81.3%) surviving to discharge (Table 8). Infant mortality (44.4%, 4/9) was markedly higher than that of non-infant (15.9%, 13/82; RD + 28.6%, 95% CI 1.3%–58.2%; exploratory *P* = 0.05). Among the novel cases, both deaths occurred within 19–30 h of admission before etiological diagnosis could be established, while Case 3 survived following early molecular diagnosis and targeted combination therapy. All 4 patients who received etoposide survived, though the low utilization (8.3%) indicates most cases were successfully managed with anti-rickettsial therapy and immunomodulation alone.

**Table 8:** Outcomes of pediatric scrub typhus-associated HLH

Outcome	All cases *N* = 91	Infants (< 12 months) *N* = 9	Non-infants (≥ 12 months) *N* = 82	RD (95% CI)	Exploratory *P* value*
Survived	74 (81.3%)	5 (55.6%)	69 (84.1%)		
Died	17 (18.7%)	4 (44.4%)	13 (15.9%)	+ 28.6% (1.3 to 58.2%)	**0.05**

^*^P values are exploratory and should not be interpreted as confirmatory, given the retrospective, heterogeneous nature of the pooled data derived from multiple countries and study designs with variable diagnostic criteria and reporting completeness

*RD* risk difference, 95% *CI* 95% confidence interval

Bold marks exploratory P values below 0.05

## Discussion

This study provides the first systematic characterization of scrub typhus-associated HLH in young infants drawing on a pooled cohort of 91 pediatric patients—the largest reported to date. Three clinically important differences distinguished infants from older children: higher mortality (44.4% vs. 15.9%), lower eschar detection (22.2% vs. 66.7%), and universal CNS involvement (100% vs. 42.9%). Together, these findings define a clinically distinct syndrome that warrants a dedicated diagnostic and therapeutic approach.

The near absence of eschar in infants constitutes the most immediate diagnostic challenge. While eschar detection rates of 60–70% are reported in pediatric series and 30–70% in adults [33, 34], only 22.2% of infants in this cohort had identifiable lesions—likely due to anatomical concealment within scalp folds, axillae, or the diaper area. This deficit carries serious clinical consequences: without this pathognomonic sign, serological confirmation requires 7–10 days for antibody development [10], a window incompatible with the fulminant course seen in our fatal cases, both of whom died within 30 h of admission. Molecular diagnostics are therefore indispensable in eschar-negative infants. In Case 3, CSF tNGS simultaneously confirmed the etiology and documented CNS invasion, directly guiding the decision of rifampin for enhanced CNS penetration.

Several factors may plausibly contribute to the higher infant mortality observed, though their independent effects cannot be disentangled without multivariable analysis. Diagnostic delay appears particularly important: the surviving infant presented after only 4 days of fever, compared with 6–11 days in fatal cases. Universal CNS involvement, immune immaturity, and co-infections (CMV, EBV) likely compound this risk, though causal attribution to scrub typhus alone cannot be established [13, 35]. An additional speculative consideration is the differential empirical antibiotic use across age groups: fluoroquinolones, commonly used in febrile adults, possess activity against O. tsutsugamushi [36], and may provide inadvertent pre-diagnostic anti-rickettsial coverage. As fluoroquinolones are avoided in children due to cartilage toxicity concerns [37, 38], this therapeutic “safety net” is absent in the pediatric setting—reinforcing the importance of early empirical doxycycline when rickettsial disease is suspected. We acknowledge this hypothesis cannot be substantiated by the current data. Similarly, the shared Lahu ethnicity and remote rural residence of our three novel cases likely contributed to delayed presentation, though the contribution of these socioeconomic factors to outcomes cannot be quantified without comparative data [39].

Regarding treatment, etoposide was used in 8.3% of cases in this cohort, and all four patients who received it survived; however, given the small number of treatment initiation, and the inherent selection bias in observational data, no inference regarding its necessity or efficacy can be drawn. The decision to use etoposide should follow institutional HLH protocols and not be delayed when clinically indicated. Case 3 illustrates that comprehensive management with doxycycline, rifampin, corticosteroids, cyclosporine, and IVIG achieved complete recovery [40]. Doxycycline remains first-line regardless of age [41, 42]; for CNS involvement, rifampin may be added for enhanced blood–brain barrier penetration [43].

**Fig. 1:**
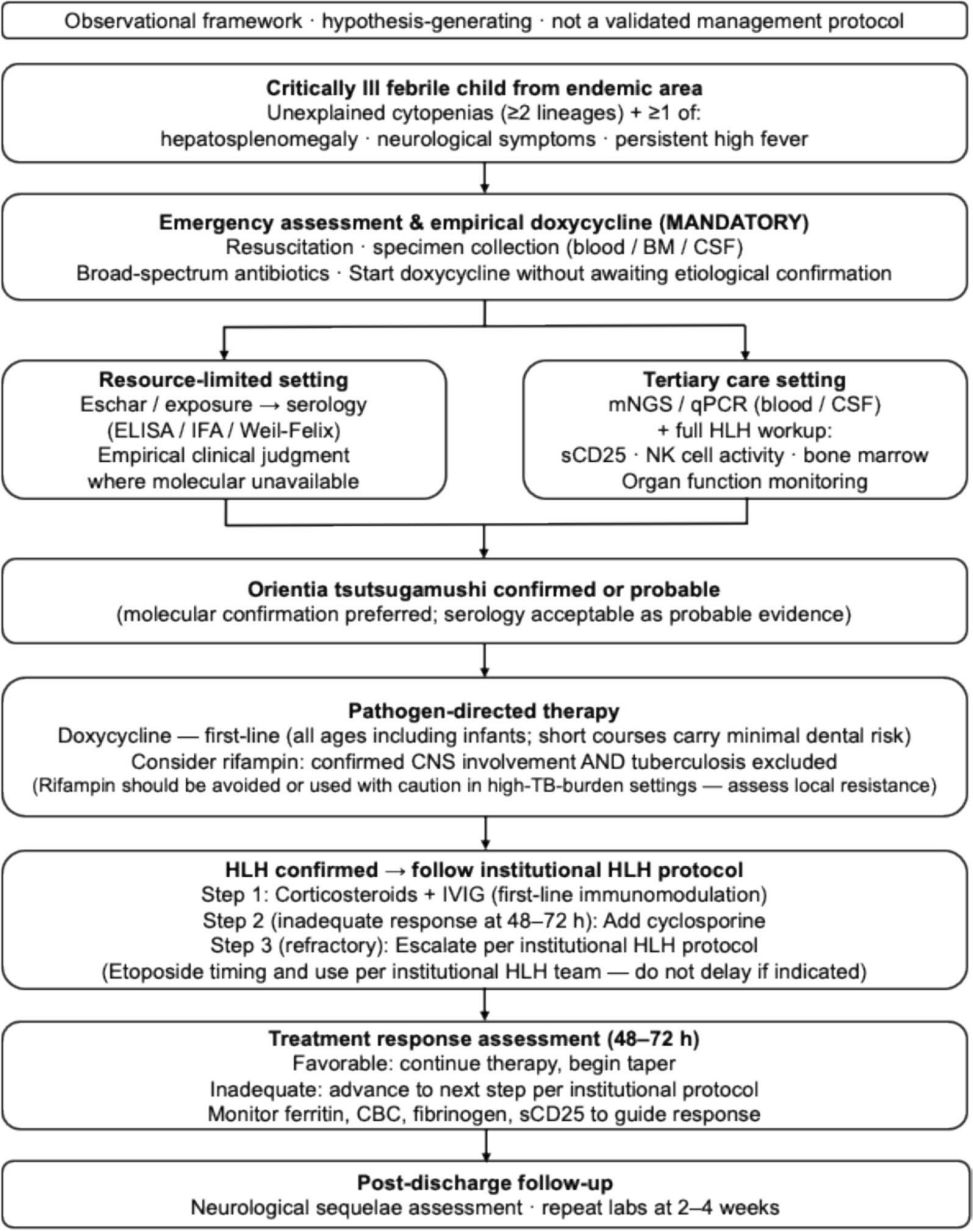
Proposed clinical decision framework for pediatric scrub typhus-associated HLH. This framework is observational, and hypothesis-generating based on 91 pooled cases. It is not a validated protocol. Treat as supplementary clinical guidance pending prospective validation

To support clinical decision-making in endemic settings, we propose a stepwise management framework (Fig. 1) integrating parallel diagnostic and therapeutic pathways. The algorithm specifies empirical doxycycline as mandatory upon clinical suspicion, irrespective of eschar status; delineates resource-stratified diagnostic approaches (serology in resource-limited settings; NGS or qPCR in tertiary centers); and immunomodulatory escalation follows institutional HLH protocols.

This study has several limitations. As a narrative pooled analysis rather than a formal systematic review, publication bias and case-level heterogeneity cannot be excluded. The infant subgroup comprised only nine cases, limiting statistical power—though this rarity itself underscores the importance of systematic characterization. Retrospective data precluded standardized HLH-2004 assessment across all cases, and co-infections may have contributed to HLH in some instances. All comparative findings are therefore exploratory and should not be interpreted as confirmatory.

In conclusion, scrub typhus-associated HLH in young infants carries high mortality due to frequent eschar absence and universal CNS involvement—a clinical profile distinct from older children. Clinicians in endemic areas should maintain heightened suspicion for this condition, prioritize molecular diagnostics in eschar-negative presentations and initiate empirical therapy without delay. Prospective multicenter studies are needed to validate these findings and establish evidence-based management guidelines for this vulnerable population.

## Supplementary Information

Supplementary Material 1.

Supplementary Material 2.

## Data Availability

The datasets used and analyzed during this study are available from the corresponding author upon reasonable request.

## Abbreviations

ALT: Alanine aminotransferase
APTT: Activated partial thromboplastin time
ARDS: Acute respiratory distress syndrome
AST: Aspartate aminotransferase
BM: Bone marrow
CARE: CAse REport guidelines
CNS: Central nervous system
CNKI: China National Knowledge Infrastructure
CRP: C-reactive protein
CRRT: Continuous renal replacement therapy
CSF: Cerebrospinal fluid
CK-MB: Creatine kinase-MB
DIC: Disseminated intravascular coagulation
EBV: Epstein–Barr virus
ELISA: Enzyme-linked immunosorbent assay
HLH: Hemophagocytic lymphohistiocytosis
IFA: Indirect fluorescent antibody
IL: Interleukin
IQR: Interquartile range
IVIG: Intravenous immunoglobulin
LDH: Lactate dehydrogenase
mNGS: Metagenomic next-generation sequencing
MODS: Multiple Organ Dysfunction Syndrome
NGS: Next-generation sequencing
NK: Natural killer
NT: Not tested
NT-proBNP: N-terminal pro-B-type natriuretic peptide
PCR: Polymerase chain reaction
PCT: Procalcitonin
PICU: Pediatric intensive care unit
PT: Prothrombin time
qPCR: Quantitative polymerase chain reaction
sCD25: Soluble interleukin-2 receptor alpha (soluble CD25)
SPSS: Statistical Package for the Social Sciences
tNGS: Targeted next-generation sequencing
WBC: White blood cell
WHO: World Health Organization
