# Supplementary material for: High infant mortality, eschar absence, and universal CNS involvement in scrub typhus-associated HLH: a case series and narrative synthesis of 91 pediatric cases
Source: Trop Med Health. 2026 May 30;54:117. doi: 10.1186/s41182-026-00988-6 (PMC13277184; doi:10.1186/s41182-026-00988-6)
Supplement: Supplementary file 1 — Supplementary Material 1. [file 41182_2026_988_MOESM1_ESM.docx]

Supplementary Table S1

STROBE Statement—checklist of items that should be included in reports of observational studies

|  | Item No. | Recommendation | Page  No. | Relevant text from manuscript |
| --- | --- | --- | --- | --- |
| **Title and abstract** | 1 | (*a*) Indicate the study’s design with a commonly used term in the title or the abstract | 1 | “a case series and narrative synthesis of 91  pediatric cases” (title); “case series” and “pooled  analysis”stated in abstract |
|  |  | (*b*) Provide in the abstract an informative and balanced summary of what was done and what was found | 1-2 | Entire abstract summarizes background, methods, results, conclusions |
| Introduction | | | |  |
| Background/rationale | 2 | Explain the scientific background and rationale for the investigation being reported | 2-4 | Background section explains scrub typhus, HLH, infant vulnerability |
| Objectives | 3 | State specific objectives, including any prespecified hypotheses | 4 | “Our objectives were to: (1) characterize clinical features… (2) identify pooled characteristics… (3) identify factors associated with mortality” |
| Methods | | | |  |
| Study design | 4 | Present key elements of study design early in the paper | 5 | “This study combines a retrospective case series with a comprehensive literature search and narrative pooled analysis” |
| Setting | 5 | Describe the setting, locations, and relevant dates, including periods of recruitment, exposure, follow-up, and data collection | 5-6 | Pu'er People's Hospital, Yunnan, China; May–Oct 2025; databases searched up to Dec 2025 |
| Participants | 6 | (*a*) *Cohort study*—Give the eligibility criteria, and the sources and methods of selection of participants. Describe methods of follow-up  *Case-control study*—Give the eligibility criteria, and the sources and methods of case ascertainment and control selection. Give the rationale for the choice of cases and controls  *Cross-sectional study*—Give the eligibility criteria, and the sources and methods of selection of participants | 5-6 | Inclusion criteria: age <18y, confirmed scrub typhus, HLH-2004 criteria |
|  |  | (*b*) *Cohort study*—For matched studies, give matching criteria and number of exposed and unexposed  *Case-control study*—For matched studies, give matching criteria and the number of controls per case | N/A | Not applicable |
| Variables | 7 | Clearly define all outcomes, exposures, predictors, potential confounders, and effect modifiers. Give diagnostic criteria, if applicable | 5-8 | HLH-2004 criteria, scrub typhus case definitions, mortality, CNS involvement, eschar |
| Data sources/ measurement | 8* | For each variable of interest, give sources of data and details of methods of assessment (measurement). Describe comparability of assessment methods if there is more than one group | *6-7* | *Electronic medical records; literature search from PubMed, Embase, CNKI, etc.* |
| Bias | 9 | Describe any efforts to address potential sources of bias | 7-8 | Independent duplicate screening and extraction;  disagreements resolved by consensus or third  investigator; duplicate case identification by  matching demographics and admission dates;  sensitivity analysis restricted to complete individual patient data |
| Study size | 10 | Explain how the study size was arrived at | 7 | “91 pediatric cases” – all available individual patient data |

Continued on next page

| Quantitative variables | 11 | Explain how quantitative variables were handled in the analyses. If applicable, describe which groupings were chosen and why | 8 | Median with IQR, categorical variables as frequencies/percentages |
| --- | --- | --- | --- | --- |
| Statistical methods | 12 | (*a*) Describe all statistical methods, including those used to control for confounding | 8 | Chi-square, Fisher’s exact, Mann-Whitney U, risk differences, exploratory p-values |
|  |  | (*b*) Describe any methods used to examine subgroups and interactions | 8 | Infants vs non-infants, survivors vs non-survivors |
|  |  | (*c*) Explain how missing data were addressed | 7-8 | Recorded as “not reported”; sensitivity analysis performed |
|  |  | (*d*) *Cohort study*—If applicable, explain how loss to follow-up was addressed  *Case-control study*—If applicable, explain how matching of cases and controls was addressed  *Cross-sectional study*—If applicable, describe analytical methods taking account of sampling strategy | N/A | Not applicable (hospital-based) |
|  |  | (*e*) Describe any sensitivity analyses | 8 | Mortality comparison restricted to complete individual patient data |
| Results | | | | |
| Participants | 13* | (a) Report numbers of individuals at each stage of study—eg numbers potentially eligible, examined for eligibility, confirmed eligible, included in the study, completing follow-up, and analysed | 9-14 | Case series (n=3) + literature cases (n=88) = 91 total |
|  |  | (b) Give reasons for non-participation at each stage | N/A | Not applicable |
|  |  | (c) Consider use of a flow diagram | N/A | Not included but not required |
| Descriptive data | 14* | (a) Give characteristics of study participants (eg demographic, clinical, social) and information on exposures and potential confounders | 15-16 | Table 3: age, sex, ethnicity, exposure |
|  |  | (b) Indicate number of participants with missing data for each variable of interest | 16-19 | Reported in tables and text (e.g., “not reported”) |
|  |  | (c) *Cohort study*—Summarise follow-up time (eg, average and total amount) | N/A | Not applicable |
| Outcome data | 15* | *Cohort study*—Report numbers of outcome events or summary measures over time | *19-20* | *Mortality, survival, complications* |
|  |  | *Case-control study—*Report numbers in each exposure category, or summary measures of exposure |  |  |
|  |  | *Cross-sectional study—*Report numbers of outcome events or summary measures |  |  |
| Main results | 16 | (*a*) Give unadjusted estimates and, if applicable, confounder-adjusted estimates and their precision (eg, 95% confidence interval). Make clear which confounders were adjusted for and why they were included | 16-20 | Risk differences, 95% CIs, exploratory p-values |
|  |  | (*b*) Report category boundaries when continuous variables were categorized | 8 | Infants <12 months, CNS involvement defined |
|  |  | (*c*) If relevant, consider translating estimates of relative risk into absolute risk for a meaningful time period | N/A | Not applicable |

Continued on next page

| Other analyses | 17 | Report other analyses done—eg analyses of subgroups and interactions, and sensitivity analyses | 8，19-20 | Sensitivity analysis, subgroup comparisons |
| --- | --- | --- | --- | --- |
| Discussion | | | | |
| Key results | 18 | Summarise key results with reference to study objectives | 20 | Higher infant mortality, lower eschar detection, universal CNS involvement |
| Limitations | 19 | Discuss limitations of the study, taking into account sources of potential bias or imprecision. Discuss both direction and magnitude of any potential bias | 23-24 | Small infant subgroup, retrospective, publication bias, missing data |
| Interpretation | 20 | Give a cautious overall interpretation of results considering objectives, limitations, multiplicity of analyses, results from similar studies, and other relevant evidence | 20-24 | All findings exploratory; no causal claims |
| Generalisability | 21 | Discuss the generalisability (external validity) of the study results | 24 | Findings applicable to endemic regions; limited by case origin |
| Other information | |  | | |
| Funding | 22 | Give the source of funding and the role of the funders for the present study and, if applicable, for the original study on which the present article is based | 26-27 | China Medical Board Grant #24-568; funder had no role |

*Give information separately for cases and controls in case-control studies and, if applicable, for exposed and unexposed groups in cohort and cross-sectional studies.

**Note:** An Explanation and Elaboration article discusses each checklist item and gives methodological background and published examples of transparent reporting. The STROBE checklist is best used in conjunction with this article (freely available on the Web sites of PLoS Medicine at http://www.plosmedicine.org/, Annals of Internal Medicine at http://www.annals.org/, and Epidemiology at http://www.epidem.com/). Information on the STROBE Initiative is available at www.strobe-statement.org.
