# Supplementary material for: High infant mortality, eschar absence, and universal CNS involvement in scrub typhus-associated HLH: a case series and narrative synthesis of 91 pediatric cases
Source: Trop Med Health. 2026 May 30;54:117. doi: 10.1186/s41182-026-00988-6 (PMC13277184; doi:10.1186/s41182-026-00988-6)
Supplement: Supplementary file 2 — Supplementary Material 2. [file 41182_2026_988_MOESM2_ESM.docx]

Supplementary Table S2. Search strategies for all databases (Search date: December 15, 2025)

| **English Databases (PubMed, Embase, Web of Science)**  The following search string was adapted for each database syntax | |
| --- | --- |
| **Concept** | **Search Terms / Keywords** |
| #1: Scrub Typhus | “scrub typhus’’ OR “Orientia tsutsugamushi” OR “tsutsugamushi” |
| #2: HLH | “hemophagocytic lymphohistiocytosis” OR “hemophagocytic syndrome” OR “HLH” OR “macrophage activation syndrome” |
| #3: Pediatric | “child” OR “infant” OR “adolescent” OR “pediatric” |
| Final Strategy | #1 AND #2 AND #3 |
| **Chinese Databases (CNKI, Wanfang Data)** | |
| **Concept** | **Search Terms (Chinese)** |
| #1: Scrub Typhus | “恙虫病” (Scrub Typhus) OR “恙虫” (Tsutsugamushi) |
| #2: HLH | “噬血细胞淋巴组织细胞增生症” (Hemophagocytic lymphohistiocytosis) OR “噬血细胞综合征” (Hemophagocytic syndrome) OR “HLH” OR “巨噬细胞活化综合征” (Macrophage activation syndrome) |
| #3: Pediatric | “儿童” (Children) OR “婴儿” (Infant) OR “青少年” (Adolescent) |
| Final Strategy | #1 AND #2 AND #3 |

Supplementary Table S3. Detailed Laboratory Findings of Three Infant Cases

| **Parameter** | **Reference Range** | **Case 1** | **Case 2** | **Case 3** |
| --- | --- | --- | --- | --- |
| **Complete Blood Count** |  |  |  |  |
| WBC (×10⁹/L) | 5.0–12.0 | 4.7–8.0 | 10.52–12.7 | 30.82–31.27 |
| Neutrophils (%) | 20–40 | 37.5–65.0 | 50.6–59.6 | 53–59 |
| Lymphocytes (%) | 50–70 | 29.4–54.6 | — | 36 |
| Hemoglobin (g/L) | 110–140 | 72–113 | 105–113 | 92–98 |
| Platelets (×10⁹/L) | 100–300 | 30–81 | 31–370 | 19–27 |
| **Inflammatory Markers** |  |  |  |  |
| CRP (mg/L) | <8 | 42.67–74.09 | 42.67–74.09 | 71.94 |
| PCT (ng/mL) | <0.5 | 1.93–5.24 | 2.12–57.6 | — |
| IL-6 (pg/mL) | <7 | 244.89–367.59 | 705 | 369 |
| IL-10 (pg/mL) | — | — | — | 282.09 |
| **Liver Function** |  |  |  |  |
| ALT (U/L) | <40 | 76 | 105 | 92 |
| AST (U/L) | <40 | 213 | 614 | 251 |
| Total bilirubin (μmol/L) | <17.1 | — | 43.2 | 9.3 |
| Albumin (g/L) | 35–50 | — | 25 | 26 |
| LDH (U/L) | 120–300 | 1261 | 6614 | — |
| **Coagulation** |  |  |  |  |
| PT (seconds) | 11–14 | — | 9.3 | >170 |
| APTT (seconds) | 25–35 | — | 23.9 | 46.5–47.0 |
| Fibrinogen (g/L) | 2.0–4.0 | Low | 2.1 | 0.22–0.31 |
| D-dimer (μg/mL) | <0.5 | — | 36.4 | 1.07–1.49 |
| **HLH-Specific** |  |  |  |  |
| Ferritin (ng/mL) | 20–200 | NT | NT | 6,800 (peak) |
| Triglycerides (mmol/L) | <1.7 | Elevated | 4.33 | 3.33–4.48 |
| sCD25 (U/mL) | <2,400 | NT | NT | 25,714 |
| NK cells (%) | 7.0–40.0 | NT | NT | 4.18 |
| **Cardiac Markers** |  |  |  |  |
| NT-proBNP (pg/mL) | <300 | 1960–3490 | 7050 | 1999 |
| CK-MB (U/L) | <25 | — | 61 | — |
| **Renal Function** |  |  |  |  |
| Creatinine (μmol/L) | 18–35 | — | 52.2 | — |
| Urea (mmol/L) | 1.8–6.4 | — | 7.05 | — |
| **Blood Gas** |  |  |  |  |
| pH | 7.35–7.45 | 7.356–7.451 | 7.21 | — |
| Lactate (mmol/L) | <2.0 | — | 12.8 | — |
| Base excess (mmol/L) | -2 to +2 | — | -12.8 | — |
| **CSF Analysis** |  |  |  |  |
| WBC (×10⁶/L) | <5 | 24 | — | 42.0 (initial) |
| Mononuclear (%) | — | 95.8 | — | 93.5 |
| Protein (mg/L) | <450 | 1000 | — | 1781 (initial) |
| Glucose (mmol/L) | 2.5–4.5 | 2.63 | — | 3.59 |

Supplementary Table S4. Laboratory Values in Pediatric Scrub Typhus-Associated HLH

| **Parameter** | **All Cases** | **Infants (<12 mo)** |
| --- | --- | --- |
| Hemoglobin, g/L, median (IQR) | 85 (65–113) | 75 (68–103) |
| Platelet, ×10⁹/L, median (IQR) | 45 (3–101) | 30 (18–78) |
| Ferritin, ng/mL, range | 587–19,706 | 1,367–>3,000 |
| Fibrinogen, g/L, range | 0.22–6.0 | 0.22–2.1 |
| Triglycerides, mmol/L, range | 1.43–13.4 | 2.29–13.4 |
| sCD25, U/mL, range | 8,367–25,714 | 25,714* |
| NK cell activity, %, range | 4.18–15.5 | 4.18* |

*Only Case 96 had sCD25 and NK cell data among infants.
